# Efficient production and multiscale characterization of amorphous granular potato starch by microwave-assisted ethanol treatment

**DOI:** 10.1016/j.fochx.2026.103923

**Published:** 2026-04-25

**Authors:** Seon-Min Oh, Seung-Hye Woo, Min Kyung Park, Joon-Young Jun, Yun-Sang Choi

**Affiliations:** Research Group of Food Processing, Korea Food Research Institute, Wanju 55365, Republic of Korea

**Keywords:** Amorphous granular starch, Microwave-ethanol treatment, Morphology, Rheology, Crystallinity, Digestibility

## Abstract

Amorphous granular starch (AGS), characterized by loss of crystalline order while retaining granular morphology, exhibits altered hydration and digestibility compared to native starch; however, conventional production methods often rely on strong alkaline reagents. This study developed a green and rapid approach to prepare AGS from potato starch using microwave-assisted ethanol treatment (55–70% *v*/v). X-ray diffraction confirmed a pronounced loss of crystalline order, with relative crystallinity decreasing from 21.81% to 3.20%. Scanning electron microscopy revealed ethanol-dependent morphology, with deformation and agglomeration at lower ethanol concentrations and higher integrity at elevated levels. Rheological properties showed reduced shear-thinning behavior and increased viscosity, indicating a shift toward Newtonian flow. Rapid visco analyzer profiles demonstrated disappearance of typical pasting behavior. *In vitro* digestion indicated that rapidly digestible starch increased from 6.12% to 26.19%, accompanied by accelerated hydrolysis kinetics. Overall, microwave–ethanol treatment provides a clean-label and efficient approach for producing AGS with tunable properties.

## Highlights

• Amorphous granular starch (AGS) was rapidly prepared using microwave–ethanol treatment.
• Granular morphology was retained while internal crystallinity was selectively disrupted.
• Structural, rheological, and digestive properties were modulated by ethanol concentration.
• MAGS showed enhanced enzymatic digestibility and shear-responsive viscosity behavior.
• The process provides a clean-label strategy for functional starch design in food systems.

## 1 Introduction

Amorphous granular starch (AGS), modified starch with a unique form, maintains its external granular shape while its losing internal crystallinity, positioning it between native starch and fully gelatinized systems. This intermediate structural state imparts distinct functional characteristics, including altered hydration behavior, dispersion properties, and enhanced starch reactivity (Fang et al., 2021; Song et al., 2017). Importantly, these properties are not solely governed by the amorphous nature itself but are strongly influenced by the processing history through which amprphization is achieved. Different modification routes-such as chemical, thermal, or physical treatments-can result in varying degrees of molecular disruption, chain scission, entanglement, and spatial distribution of amorphous domains leading g to divergent functional behaviors even among starches classified as amorphous. Conventional AGS production typically relies on strongly alkaline reagents. For example, Choi, Baik, and Kim (2017) prepared amorphous starch *via* ethanol treatment followed by incremental addition of NaOH, while Chen et al. (2019) and Kaveh, Azadmard-Damirchi, Yousefi, and Hosseini (2020) employed highly alkaline aqueous or ethanol–alkali systems to disrupt crystalline structures. Although effective in reducing crystallinity, such chemical approaches may induce excessive swelling, molecular degradation, and partial loss of granular integrity, and typically require multi-step processing. Moreover, depending on treatment conditions, alcoholic–alkaline systems can generate diverse structural outcomes, including partial recrystallization or V-type complex formation (Majzoobi, Kaveh, Blanchard, & Farahnaky, 2015; Zhang, Dhital, Haque, & Gidley, 2012). These considerations suggest the need for alternative physical strategies capable of inducing controlled amorphization while preserving granular morphology.

Microwave-assisted modification has emerged as an environmentally compatible alternative owing to its rapid and volumetric heating mechanism driven by dielectric polarization (Wei, Li, & Zhu, 2025). Unlike conventional conductive hating, microwave energy interacts directly with polar components such as water, enabling efficient energy transfer throughout the starch matrix. As a result, microwave-treated starches frequently exhibit alteration in crystallinity, swelling behavior, and digestibility, depending on processing parameters including moisture availability and treatment intensity (Palav & Seetharaman, 2007; Wang, Wang, Zhou, Wu, & Ouyang, 2022; Ye & Baik, 2024). When ethanol is incorporated as a co-solvent, its lower polarity reduces water accessibility, thereby moderating excessive swelling and facilitating amorphization without the use of chemical reagents. This combined approach offers a cleaner and faster route for amorphous starch preparation; however, systematic investigations linking microwave–ethanol processing conditions to multiscale structural and functional properties remain limited. Therefore, this study aimed to prepare potato AGS through a microwave–ethanol process and to evaluate its structural, thermal, rheological, and digestibility characteristics. The findings are expected to provide insight into the physicochemical transformations and functional potential of starch modified through solvent-assisted microwave treatment.

## 2 Materials and methods

### 2.1 Materials

Commercial potato starch was purchased from Ever Health Care Foods (Icheon, South Korea). Its proximate composition (wet basis) was as flows: moisture 15.50%, ash 0.30%, protein 0.22%, and lipid 0.34%. Total starch content (94.1%, dry basis) was determined using Total starch assay kit (Meagazyme, Wicklow, Ireland), and amylose content (20.7%) was analyzed using colorimetric method (Oyeyinka, Akintayo, Adebo, Kayitesi, & Njobeh, 2021). Ethanol (95% purity) was obtained from Ethanol Supplies World Co., Ltd. (Jeonju, South Korea). All other chemicals and reagents used in this study were of analytical grade.

### 2.2 Preparation of microwave-assisted AGS (MAGS)

The ethanol concentration range was determined based on preliminary screening experiments. At lower ethanol levels, microwave treatment resulted in complete gelatinization and collapse of granular structure, whereas higher concentrations facilitated the formation of amorphous starch while retaining granular features. The selected range was therefore used to investigate the structural transition from fully gelatinized to amorphous granular states, with 63% representing a transitional condition. The microwave conditions were selected based on preliminary experiments to ensure sufficient structural modification within a short treatment time while avoiding excessive degradation.

Thirty grams (d.b.) of potato starch was mixed with 100 mL of ethanol solution at varying concentrations (55%, 60%, 63%, 65%, and 70% *v*/v) in a microwave-compatible polypropylene (PP) autoclave container (SPL Life Sciences, Pochen, Korea; 120 × 80 mm; internal dimensions 103 × 78.6 mm). The lid was loosely placed to prevent pressure build-up during microwave treatment. The mixture was then exposed to microwave irradiation (MW25S; LG Electronics Tianjin Appliance Co., Ltd., China) at 2450 MHz and 700 W for 1 min. Immediately after microwave treatment, an additional 100 mL of ethanol was added to promote dehydration and suppress further swelling. The suspension was centrifuged at 4000 rpm for 10 min, and the obtained MAGS was dried in a drying oven at 30 °C. The dried samples were ground and passed through an 80-mesh sieve before analysis. Native potato starch (NPS) was used as a control, and microwave-assisted AGS (MAGS) were designated as MAGS-55, MAGS-60, MAGS-63, MAGS-65, and MAGS-70, according to the ethanol concentration (*v*/v).

### 2.3 Microscopic analysis

The morphological characteristics of the starch samples were observed by light microscopy, polarized light microscopy, and field-emission scanning electron microscopy (FE-SEM). For light and polarized light microscopy, starch was dispersed in distilled water at a concentration of 1% (*w*/*v*), and a drop of the suspension was mounted on a glass slide and covered with a coverslip for observation. Images were captured using a light microscope (BX40; Olympus Co., Tokyo, Japan) equipped with polarized light filters to observe the birefringence patterns.

For FE-SEM, the samples were mounted on aluminum stubs using double-sided carbon tape and coated with a thin layer of platinum using a sputter coater to enhance conductivity. Surface morphology was observed using a field-emission scanning electron microscope (S-4700, Hitachi, Japan) at an accelerating voltage of 10 kV.

### 2.4 Particle size analysis

The particle sizes of the samples were measured using a Cilas 1190 laser diffraction particle size analyzer (Cilas, Orleans, France) following the method described by Kim, Rahman, Lee, and Choi (2021). The sample was poured into the dispersion circulator tank of the analyzer containing distilled water and sufficiently dispersed with water until the obscuration value reached 12%. The D0.1, D0.5, and D0.9 values represent the particle diameters below which 10%, 50%, and 90% of the cumulative particle volume was determined, respectively. The mean particle diameter was calculated from the volume-weighted average.

### 2.5 X-ray diffraction (XRD) analysis

The crystalline structures of the NPS and MAGS samples were analyzed using an X-ray diffractometer (SmartLab; Rigaku, Tokyo, Japan). Measurements were conducted over a 2θ range of 4–40°, with a scanning rate of 6°/min and a step size of 0.02°. The X-ray was operated at 45 kV and 200 mA. The relative crystallinity of the starch samples was calculated as the ratio of crystalline and amorphous regions in the diffractograms.

### 2.6 Differential scanning calorimetry (DSC)

The thermal properties of the starch samples were analyzed using a differential scanning calorimeter (DSC 4000, PerkinElmer, Waltham, MA, USA). The starch samples were mixed with distilled water to prepare a suspension with 75% moisture content (*w*/w). The suspension was vortex-mixed thoroughly and equilibrated for 1 h at room temperature to ensure uniform hydration. Immediately before sampling, the suspension was gently stirred to prevent sedimentation, and 10 mg of the homogeneous mixture was weighed and sealed in a stainless-steel pan. Ten milligrams of the suspension was sealed in a stainless-steel pan, with an empty pan used as a reference. The samples were scanned from 30 to 120 °C at a heating rate of 10 °C/min under a nitrogen atmosphere. The onset temperature (To), peak temperature (Tp), conclusion temperature (Tc), and gelatinization enthalpy (ΔH) were determined.

### 2.7 Rapid Visco Analyzer (RVA) analysis

The pasting properties of the starch samples were analyzed using a RVA (RVA Super 4; Newport Scientific, Australia). A sample suspension (3.0 g starch in 25 mL of distilled water) was adjusted to a moisture content of 14% (wet basis) prior to analysis. RVA measurements were performed following the Standard 1 profile: the samples were equilibrated at 50 °C for 1 min, heated to 95 °C at a rate of 6 °C/min, held at 95 °C for 5 min, then cooled to 50 °C at the same rate, and finally held at 50 °C for 2 min.

### 2.8 Solubility and swelling power

The solubility and swelling power of the starch samples were determined using a modified method from a previous study (Oh et al., 2019). Briefly, 0.5 g of starch (d. b) was suspended in 30 mL distilled water in a pre-weighed centrifuge tube. The suspension was incubated in a water bath at 30 °C or 60 °C for 30 min. After incubation, the samples were centrifuged at 2258 ×*g* for 20 min. The supernatant was carefully separated and dried at 105 °C to determine the amount of solubilized solids. The weight of the swollen sediment remaining in the tube was recorded to determine swelling power.

$$\mathrm{Solubility}\;\left(\%\right)=\frac{\mathrm{Solid weight}\;\left(g\right)\;\mathrm{in supernatant after drying}}{\mathrm{Initial sample weight}\;\left(g\right)}\times 100$$

$$\mathrm{Swelling power}\;\left(g/g\right)=\frac{\mathrm{Sediment weight}\;\left(g\right)\times 100}{\mathrm{Initial sample weight}\;\left(g\right)\times \left(100-\mathrm{solubility}\%\right)}$$

### 2.9 Rheological properties

The steady shear rheological properties of the starch suspensions were analyzed using a rotational rheometer (MCR 102, Anton Paar GmbH, Graz, Austria) with a parallel plate geometry (diameter 40 mm, gap 1 mm). Each sample was prepared as 10% (*w*/*v*) starch suspension in distilled water and equilibrated at room temperature before measurement. Flow behavior was determined over shear rates ranging from 0.1 to 100 s^−1^ at 25 °C. A power-law regression was fitted to characterize the flow behavior and describe the relationship between the shear rate and shear stress.

$$\tau =K\cdot \gamma^{n}$$

where τ represents shear stress (mPa), γ is the shear rate (s^−1^), *K* is the consistency coefficient (mPa·s^n^), and *n* is the flow behavior index.

### 2.10 *In-vitro* digestibility

The *in vitro* digestibility of NPS and MAGS was evaluated using a modified method described by Wang, Zhao, Wu, Wang, and Ouyang (2020). Each sample (100 mg) was suspended in 4 mL of an enzymatic digestion solution containing pancreatic α-amylase (3 CU/mg) and amyloglucosidase (3300 U/mL), followed by incubation in a shaking water bath at 37 °C. Aliquots (100 μL) were collected at designated time intervals (0, 10, 20, 30, 40, 60, 90, 120, and 150 min) and immediately mixed with 900 μL of absolute ethanol to terminate enzymatic activity. The mixtures were centrifuged at 2258 ×*g* for 10 min, and 100 μL of the supernatant was reacted with 3 mL of glucose oxidase-peroxidase (GOPOD) reagent at 50 for 20 min. The absorbance was measured at 510 nm using a spectrophotometer, with a reagent blank as a reference. Based on the hydrolysis profiles, the starch fractions were categorized as rapidly digestible starch (RDS; hydrolyzed within 20 min), slowly digestible starch (SDS; hydrolyzed between 20 and 120 min), and resistant starch (RS; remaining undigested after 120 min). The hydrolysis kinetics were calculated using the following equations:

C=C∞∙1−e^−kt

where *C* is the percentage of starch digested, *C*_*∞*_ is the equilibrium hydrolysis percentage of hydrolyzed sample, and *k* is the digestion rate constant (min^−1^).

### 2.11 Statistical analysis

All experiments were conducted in triplicate, and the results are presented as mean ± standard deviation. Statistical analysis was performed using SPSS software (ver.20, IBM Corp., Armonk, NY, USA). Multi-factor analysis (MFA) was carried out using R software. One-way analysis of variance (ANOVA) was used to determine significant differences among the sample groups. Tukey's post-hoc test was used for multiple comparisons at a 95% confidence level.

## 3 Results and discussion

### 3.1 Morphological properties

**Fig. 1:**
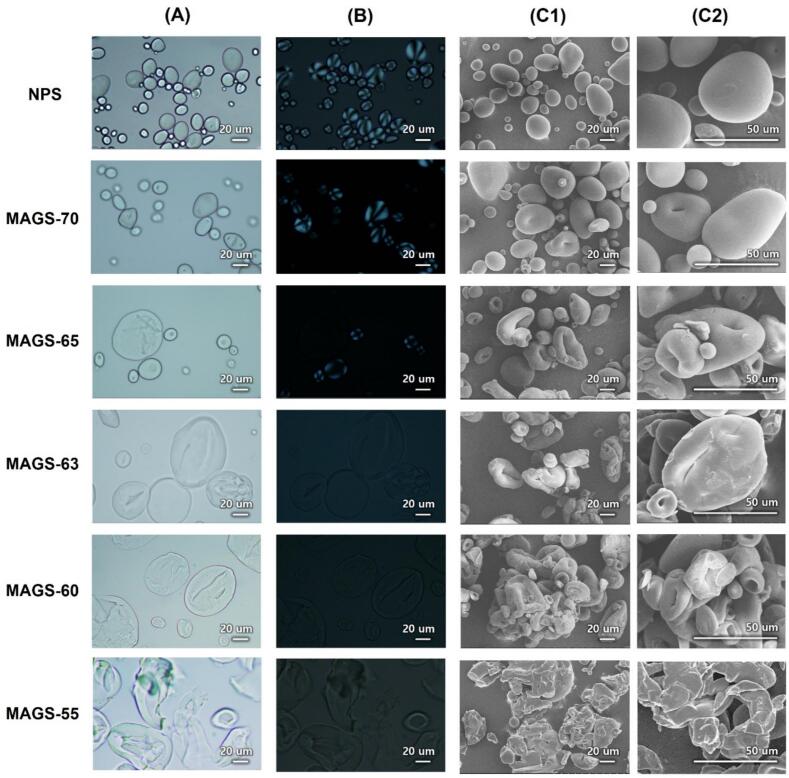
Microscopic images of MAGS prepared with different ethanol concentrations. Light microscopy (A), polarizing microscopy (B), and scanning electron microscopy at low and high magnifications (C1 and C2, respectively). scanning electron microscopy (C_1_, C_2_). SEM images were obtained at 500× (C_1_) and 1000× (C_2_) magnifications.

Polarized light microscopy revealed distinct differences in birefringence between native and microwave–ethanol–treated starches. Native potato starch (NPS) granules displayed the typical Maltese cross pattern, indicating a well-ordered radial alignment of crystalline lamellae (Butt, Ali, & Hasnain, 2019). Upon microwave–ethanol treatment, the optical anisotropy gradually weakened and eventually disappeared as the ethanol concentration decreased, reflecting the progressive loss of crystallinity and transition toward an amorphous state (Li, Qi, Li, Chen, & Xu, 2022; Wu et al., 2019). According to previous studies (Kim et al., 2017; Wang et al., 2022), potato starch granules are typically round or oval, with smooth surfaces. As shown in Fig. 1A and Fig. 1B, this morphological trait was observed in NPS. In contrast, MAGS exhibited somewhat different characteristics. For instance, MAGS-70 showed partially swollen granules with a reduced Maltese cross, suggesting that some granules were partially gelatinized without complete disruption. As the ethanol concentration decreased from 65% to 60%, the apparent granule size increased, which can be attributed to enhanced water availability and swelling under reduced ethanol conditions. Previous studies have reported that decreasing ethanol concentrations promote granule swelling and surface rupture, resulting in a slight increase in apparent granule size (Choi et al., 2017; Sarifudin, Soontaranon, Rugmai, & Tongta, 2019). The number and clarity of Maltese crosses observed under polarized light were significantly diminished, and birefringence was no longer detectable at ethanol concentrations of 63–60%, indicating a loss of crystallinity.

FE-SEM micrographs (Fig. 1C_1_-C_2_) confirmed that the surface roughness and indentation depth increased with decreasing ethanol concentration, while inter-granular adhesion became more apparent. These features indicate that microwave dielectric heating induced internal disruption of crystalline domains, whereas the ethanol-rich continuous phase suppressed excessive external swelling. These surface deformations are attributed to granule expansion induced by heating, followed by shrinkage during drying as moisture evaporates (Chen, Dai, & Gao, 2020; Kim et al., 2017). Li et al. (2022) suggested that decreasing ethanol concentrations led to the formation of cracks and minor depressions on the starch surface, potentially affecting its specific surface area.

At an ethanol concentration of 55%, granules were completely collapsed into a continuous gel-like matrix with indistinct boundaries, corresponding to extensive water penetration and full gelatinization. Clear retention of granular morphology was primarily observed at 65–70% ethanol, whereas 60–63% represented a transitional amorphization range with partial deformation. Higher ethanol levels limited dielectric coupling and water mobility, resulting in incomplete amorphization, whereas lower ethanol levels reduced this inhibitory effect, leading to excessive swelling and collapse. This finding agrees with the report of Zhang et al. (2012), who observed that starch granules dispersed in ∼66% ethanol exhibited peripheral birefringence associated with partial swelling, whereas lower ethanol ratios caused complete loss of order and granule disintegration. In NaOH-based systems, hydroxide ions penetrate the granule from the exterior, promoting outside-in gelatinization and surface erosion accompanied by chain hydrolysis. In contrast, microwave–ethanol treatment involves volumetric dielectric heating, where electromagnetic energy is directly converted into thermal energy within the material. This heating mechanism differs fundamentally from conventional conduction heating, which relies on external heat transfer and temperature gradients propagating from the surface inward. Microwave irradiation induces rapid heating and distinct gelatinization behavior in starch systems compared to conduction heating (Bilbao-Sáinz, Butler, Weaver, & Bent, 2007). In addition, dielectric polarization contributes to molecular mobility and structural rearrangement within starch granules (Fan et al., 2017; Tao et al., 2020). However, local temperature variations can arise due to differences in dielectric properties, water distribution, and ethanol concentration within the system. In particular, regions with higher water content are expected to exhibit greater microwave absorption and heat generation, whereas ethanol-rich regions, with lower dielectric loss, may suppress microwave energy absorption, consistent with the strong dependence of dielectric properties on moisture distribution within the system (Tao et al., 2020). This contrast in dielectric behavior can lead to localized heating and the formation of thermal gradients at the microscale, driven by coupled heat and mass transfer and spatial variations in moisture content (Wu et al., 2025). As a result, the disruption of crystalline domains occurs in a spatially heterogeneous manner rather than through a uniform transformation. These findings suggest that the structural modification observed in this study is governed by ethanol-modulated water availability and localized dielectric heating effects, rather than a preferential core-initiated or surface-driven mechanism.

### 3.2 Particle size

The particle size distribution and mean diameters of NPS and MAGS samples treated with microwave-ethanol are presented in Table 1. Particle sizes were characterized by measuring the D0.1, D0.5, and D0.9 values, representing diameters below which 10%, 50%, and 90% of the particle volumes were distributed, respectively. NPS exhibited a mean diameter of 39.95 ± 0.29 μm, with D0.1, D0.5, and D0.9 values of 15.64 ± 0.25 μm, 39.83 ± 0.23 μm, and 63.12 ± 0.29 μm, respectively. MAGS samples displayed notably increased particle sizes as the ethanol concentration decreased. Specifically, the mean particle diameter significantly increased from 40.37 ± 0.25 μm for MAGS-70 to 57.10 ± 0.14 μm for MAGS-55 (*p* < 0.05). This trend of increasing particle diameter with decreasing ethanol concentrations was consistently observed across all measured parameters (D0.1, D0.5, and D0.9). These results are consistent with the morphological observations (Fig. 1), where MAGS samples exhibited surface swelling, agglomeration, and partial disruption of granular integrity, particularly under at lower ethanol concentrations.

**Table 1:** Particle size and mean diameter of MAGS prepared with different ethanol concentrations.

Sample	D0.1 (**μ**m)	D0.5 (**μ**m)	D0.9 (**μ**m)	Mean diameter (**μ**m)
NPS	15.64 ± 0.25^c^	39.83 ± 0.23^e^	63.12 ± 0.29^e^	39.95 ± 0.29^e^
MAGS-70	15.97 ± 0.05^c^	40.50 ± 0.18^d^	63.27 ± 0.63^e^	40.37 ± 0.25^e^
MAGS-65	14.61 ± 0.09^d^	45.94 ± 0.06^c^	75.57 ± 0.15^d^	46.17 ± 0.05^c^
MAGS-63	15.69 ± 0.12^c^	52.29 ± 0.44^b^	90.85 ± 0.71^c^	53.25 ± 0.73^b^
MAGS-60	16.52 ± 0.08^b^	54.89 ± 0.20^a^	97.83 ± 0.09^b^	56.67 ± 0.11^a^
MAGS-55	17.13 ± 0.04^a^	54.40 ± 0.07^a^	99.24 ± 0.39^a^	57.10 ± 0.14^a^

*Values are expressed as mean ± standard deviation.

*Different letters within the same column indicate significant difference (*p* < 0.05).

Similar findings were reported by Fang et al. (2021), where AGS showed significantly larger mean particle sizes (D0.5: 30.6–57.0 μm) than native starches (D0.5: 13.3–24.6 μm). Zhang et al. (2017) suggested that the increase in granule size has been attributed to hydrogen bonding between water molecules and the exposed hydroxyl groups of starch chains, which promotes water absorption and swelling. However, in the present microwave–ethanol system, the increase in apparent particle size appears to be more closely associated with granule surface disruption, inter-particle aggregation, and increased cohesion following amorphization rather than simple reversible swelling of intact granules. The extent of granule enlargement in AGS is reported closely associated with the granule size of the native starch. For example, alcohol–alkali treatments have been reported to induce more pronounced surface deformation in potato starch than in corn starch (Zhang et al., 2012). Singh and Singh (2003) similarly reported that potato starch granules exhibited extensive surface indentations and structural irregularities in water–alcohol systems. These observations support the present findings that microwave–ethanol treatment induces notable changes in granule morphology and apparent particle size, particularly at lower ethanol concentrations. The larger inherent granule size and high hydration capacity of potato starch may further contribute to its susceptibility to aggregation and structural deformation under these conditions.

### 3.3 Crystalline structure

**Fig. 2:**
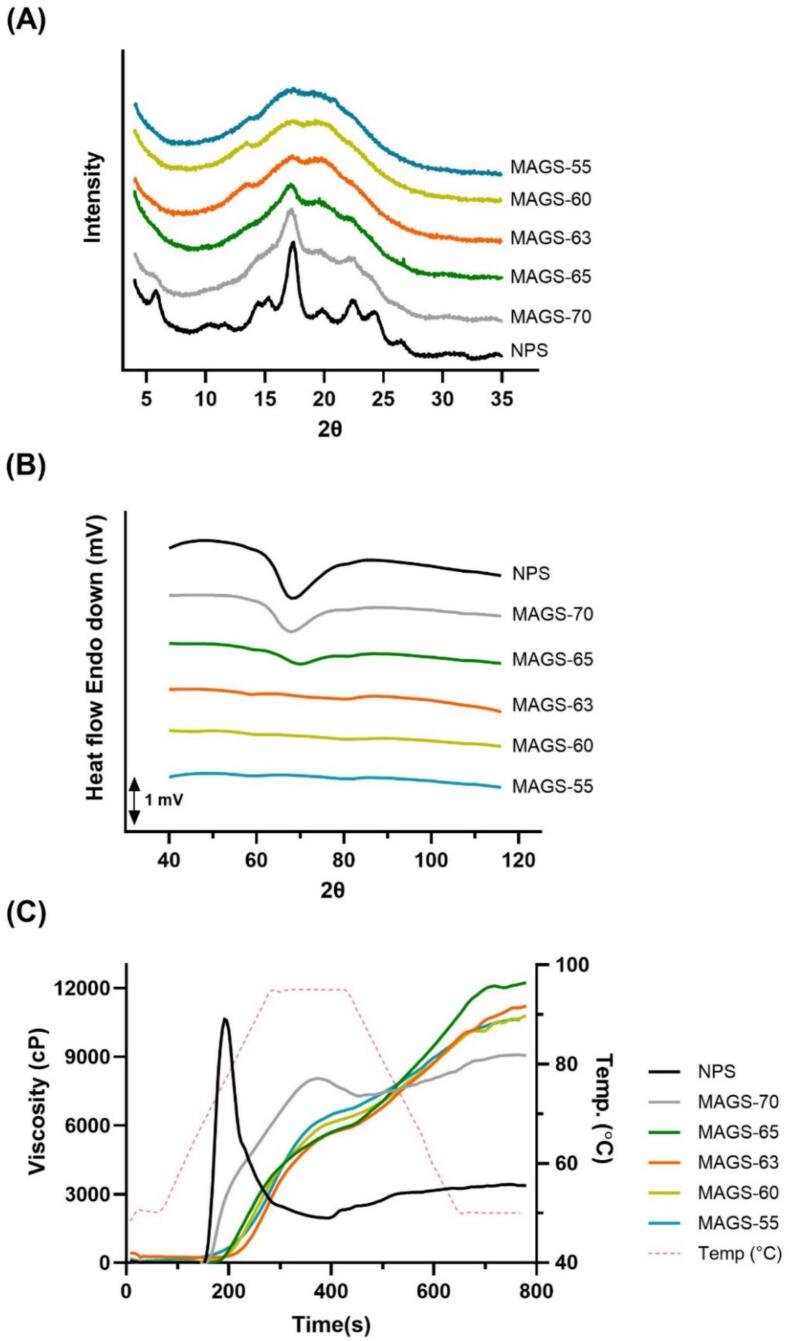
XRD patterns (A), DSC thermogram (B) and RVA profile (C) of MAGS prepared with different ethanol concentrations.

XRD profiles and relative crystallinity (RC) values are showed in Fig. 2A and Table 2. NPS exhibited distinct diffraction peaks at 5°, 15°, 17°, and 23–25° (2θ), indicative of a B-type crystalline polymorph. These sharp and well-defined peaks are representative of a long-range crystalline structure, characterized by periodic arrangement of amylopectin double helices within the granule matrix (Ma, Yin, Chang, Hu, & Boye, 2018; Shrestha et al., 2010). The RC of native starch was 21.81%, which is consistent with previously reported values for potato starch (Colussi et al., 2020; Li et al., 2016).

**Table 2:** Thermal properties and relative crystallinity of MAGS prepared with different ethanol concentrations.

Sample	RC (%)	To (°C)	Tp (°C)	Tc (°C)	ΔH (J/g dried sample)
NPS	21.81 ± 0.44^a^	62.68 ± 0.14^a^	68.45 ± 0.16^b^	79.84 ± 1.02^a^	17.32 ± 0.30^a^
MAGS-70	10.91 ± 0.51^b^	61.28 ± 0.48^b^	67.61 ± 0.31^c^	77.55 ± 0.80^b^	12.53 ± 1.97^b^
MAGS-65	6.86 ± 0.26^c^	61.41 ± 0.40^b^	69.60 ± 0.28^a^	77.83 ± 0.30^ab^	5.76 ± 0.50^c^
MAGS-63	4.83 ± 0.31^d^	55.42 ± 0.71^c^	58.80 ± 0.24^d^	63.00 ± 0.99^c^	0.40 ± 0.06^d^
MAGS-60	3.34 ± 0.03^e^	N·D	N·D	N·D	N·D
MAGS-55	3.20 ± 0.21^e^	N·D	N·D	N·D	N·D

*Values are expressed as mean ± standard deviation.

*Different letters within the same column indicate significant difference (*p* < 0.05).

*RC: relative crystallinity; To: onset temperature; Tp: peak temperature; Tc: conclusion temperature (°C); ΔH: gelatinization enthalpy.

After microwave–ethanol treatment, the intensity of these diffraction peaks markedly decreased, particularly at 5° and 23–25° (2θ), indicating a progressive loss of long-range order. The presence of a single broad peak near 17° in some treated samples (MAGS-70 and MAGS-65) suggested a residual, although significantly diminished, long-range order. As the ethanol concentration decreased from 70% to 55%, the RC values sharply declined from 10.91% to 3.20%, reflecting a progressive disruption of crystalline domains and the associated loss of double helical structures. This trend is consistent the findings of Zhang et al. (2012), who observed the disappearance of characteristic B-type crystalline peaks in potato starch treated with ethanol–water mixtures (starch:water:ethanol = 1:3:6). Additionally, the very low RC values (3.20–3.34%) observed in MAGS-60 and MAGS-55 are in agreement with previous reports describing such levels as indicative of highly amorphous structures (Fang et al., 2021).

Interestingly, the weak diffraction peaks observed near 13° and 20° (2θ) in MAGS-63, MAGS-60, and MAGS-55 samples may indicate the formation of V-type crystalline structures, which are often associated with amylose-ethanol complexes (Gutiérrez & Tovar, 2021). Choi et al. (2017) also reported V-type peaks corresponding to single-helical amylose–ligand interactions. These results suggest that limited molecular rearrangement may occur during treatment, leading to minor V-type crystallite formation within an otherwise amorphous matrix. From a mechanistic viewpoint, the rapid loss of B-type crystallinity can be attributed to the dielectric-driven internal heating characteristic of microwave irradiation. Unlike hydrothermal or NaOH-induced amorphization, which progresses from the exterior inward through conductive or chemical pathways, microwave energy deposits directly within water-rich lamellae inside the granule, generating localized melting of double-helical regions. Simultaneously, the ethanol-rich external phase restricts water diffusion and swelling, thereby maintaining the outer morphology and preventing complete collapse. The reduced RC may therefore reflect internal lamellar disruption under constrained external conditions, consistent with the morphological and DSC results.

### 3.4 Thermal properties

DSC was performed to investigate the thermal transitions and crystalline integrity of starch samples to complement the morphological observations described above. The DSC thermograms and related parameters are summarized in Fig. 2B and Table 1, respectively. NPS exhibited an onset temperature of 62.68 °C and a conclusion temperature of 79.84 °C, with an enthalpy of gelatinization (ΔH) of 17.32 J/g, indicating a highly ordered crystalline structure. These thermal characteristics are consistent with previous reports for native starches and correlate well with the strong birefringence and intact granule morphology observed under polarized light microscopy (Muñoz, Pedreschi, Leiva, & Aguilera, 2015). MAGS displayed progressive reductions in ΔH and narrowing of the gelatinization range, reflecting the gradual loss of crystallinity with decreasing ethanol concentration. For instance, MAGS-70 retained approximately 72% of the native ΔH (12.53 J/g), while MAGS-65 exhibited a sharp decline to 5.76 J/g, indicating substantial disruption of crystalline lamellae. The onset temperature also decreased with decreasing ethanol concentration, consistent with enhanced water accessibility and partial weakening of hydrogen-bonding networks inside the granules. At 63% ethanol, ΔH dropped to 0.40 J/g with a narrow endothermic range (55.4–63.0 °C), suggesting that only trace amounts of crystalline order remained, in agreement with the near-complete disappearance of birefringence. No endothermic peaks were observed for MAGS-60 and MAGS-55, confirming the complete loss of crystallinity and transformation to an amorphous state. Interestingly, while MAGS-60 retained its granular morphology, MAGS-55 formed a gel-like matrix. This morphological distinction suggests that amorphization may result in two distinct physical states: morphology-preserved amorphous granules and collapsed amorphous matrices.

These distinct thermal transitions highlight a non-equilibrium amorphization process induced by microwave–ethanol treatment. Microwave irradiation generates volumetric dielectric heating, causing rapid, localized melting of crystalline regions within the granule interior before overall thermal equilibrium is established. Meanwhile, the ethanol-rich medium likely limits excessive macroscopic swelling by reducing water availability. Notably, MAGS-60 and MAGS-63 exhibited no detectable gelatinization endotherm and lacked birefringence, confirming the loss of crystalline order. However, SEM images revealed that overall granule morphology was largely retained. This combination suggests that crystalline lamellae were disrupted while the macroscopic granular framework remained partially intact. In contrast, NaOH- or hydrothermal-based amorphization proceeds *via* outside-in gelatinization and chemical hydrolysis of the starch matrix, resulting in chain scission and irreversible gel formation. The rapid amorphization observed suggests efficient volumetric heating under microwave irradiation. Compared with conventional hydrothermal or alkaline treatments requiring prolonged heating (30–120 min at 80–95 °C) (Kim et al., 2017; Zhang et al., 2017), the present system achieved substantial structural modification within 1 min. These findings indicate that microwave–ethanol treatment provides a rapid and chemically mild approach for inducing amorphization.

### 3.5 Pasting properties

The pasting behavior of NPS and MAGS was assessed using a RVA, and the results are presented in Fig. 2C and Table 3. NPS displayed a typical pasting profile of semi-crystalline starch, with a distinct peak viscosity (10,593 ± 169 cP), followed by a notable breakdown (8716 ± 284 cP). This behavior reflects the well-ordered lamellar crystalline structures within native granules, which undergo swelling and gelatinization upon heating, followed by structural collapse under shear and thermal stresses. In contrast, the MAGS sample exhibited markedly different pasting profiles, indicating that the structural transformation was induced by the microwave-ethanol treatment. Specifically, MAGS-70 retained some distinguishable peaks and viscosity breakdown, while MAGS-63, MAGS-60, and MAGS-55 exhibited a continuous increase in viscosity without a classical peak or trough region. This suggests that the conventional gelatinization-peak-breakdown sequence was disrupted. The absence of sharp transitions implies that microwave–ethanol pretreatment induced partial amorphization and internal dehydration, loosening the granular matrix and diminishing the cooperative melting behavior typically associated with crystalline lamellae. These features led to gradual and sustained water uptake during heating rather than the rapid swelling observed in native starch (Vicente et al., 2025).

**Table 3:** Pasting properties of MAGS prepared with different ethanol concentrations.

Sample	Peak viscosity (cP)	Breakdown (cP)	Final viscosity (cP)	Setback (cP)	Pasting temp. (°C)
NPS	10,593 ± 169^a^	8716 ± 284^a^	3372 ± 14^e^	1496 ± 130^b^	68.6 ± 0.1^c^
MAGS-70	7953 ± 123^b^	875 ± 143^b^	8970 ± 106^d^	1892 ± 161^a^	70.2 ± 0.0^c^
MAGS-65	5895 ± 39^d^	N·D	12,254 ± 45^a^	N·D	74.3 ± 0.0^b^
MAGS-63	5678 ± 178^d^	N·D	11,008 ± 160^b^	N·D	77.5 ± 1.2^a^
MAGS-60	6086 ± 141^d^	N·D	10,543 ± 181^c^	N·D	73.5 ± 0.0^b^
MAGS-55	6602 ± 1^c^	N·D	10,593 ± 16^c^	N·D	67.7 ± 1.2^c^

*Values are expressed as mean ± standard deviation.

*Different letters within the same column indicate significant difference (*p* < 0.05).

Despite lacking the conventional peak viscosity, these samples exhibited a steady increase in viscosity throughout the heating and subsequent cooling phases, reaching high final viscosities of 10,543 cP for MAGS-60 and 11,008 cP for MAGS-63. Although this sustained viscosity increase may resemble classical setback behavior associated with amylose retrogradation, it may also reflect additional contributions from partial amorphization and structural reorganization, particularly in potato starch systems characterized by high swelling capacity and rapid amylose reassociation. The observed behavior likely arises from multiple concurrent factors, including partial amorphization, reassociation of solubilized amylose during cooling, and structural reorganization of disrupted granules. This system appears to behave as a weak but cohesive structure formed through intermolecular associations among partially disordered starch chains. Kaul, Kaur, Kaur, Mehta, and Kennedy (2023) and Li, Capuano, and Tong (2025) reported that ethanol–aqueous heat-treated potato starches exhibited negative breakdown values, resulting from enhanced structural resistance to shear and ongoing viscosity development during prolonged heating. In addition, amorphous granular starches prepared by non-thermal and thermal gelatinization have been shown to exhibit continuous viscosity buildup despite the loss of birefringence, highlighting the contribution of retained granular frameworks to rheological stability (Ye & Baik, 2024). Taken together, these results suggest that microwave–ethanol treatment induces extensive amorphization while retaining sufficient structural cohesion to support progressive viscosity development under shear, without requiring complete granular integrity or classical retrogradation as the sole mechanism. In contrast, NaOH-treated cold-water-soluble starches typically exhibit negligible peak viscosity and markedly reduced final viscosity due to extensive molecular dissociation and loss of granular integrity (Choi et al., 2017). Alkali-induced amorphization often leads to rapid solubilization of starch chains and limited viscosity development during heating and cooling, despite substantial crystallinity loss. The sustained viscosity increase observed in MAGS-60 and MAGS-63 therefore suggests that, unlike alkaline systems, microwave–ethanol treatment promotes controlled amorphization while preserving sufficient structural cohesion to support progressive network formation under shear.

### 3.6 Solubility and swelling power

**Fig. 3:**
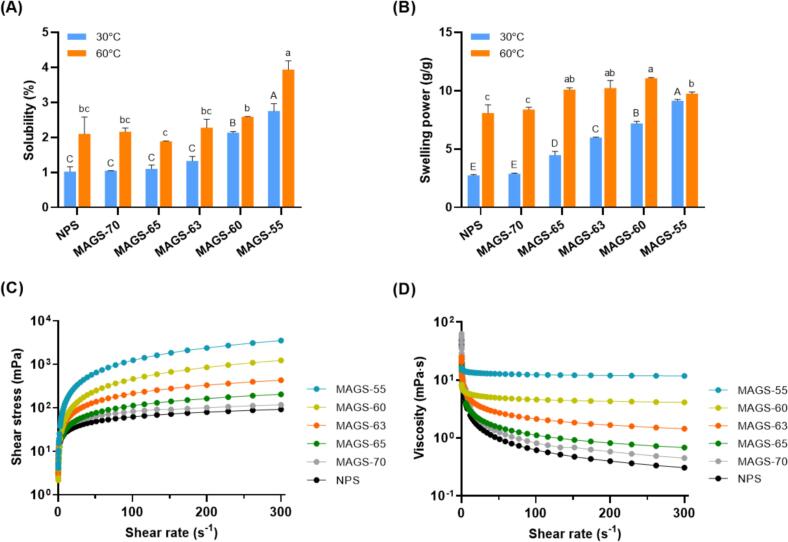
Hydration behavior and rheological properties of native potato starch (NPS) and microwave–ethanol-treated starches (MAGS); Solubility (A), swelling power (B), shear stress–shear rate (C), and viscosity–shear rate curves (D).

The solubility and swelling power of NPS and MAGS were evaluated at 30 °C and 60 °C to determine the impact of structural modification on water interaction behavior (Fig. 3A and B). At 30 °C, NPS and partially amorphous MAGS samples (MAGS-70, MAGS-65, and MAGS-63) exhibited low solubility (1.0–1.3%), with no significant difference among them. MAGS-60 showed a moderate increase of 2.13%, while fully amorphous MAGS-55 exhibited the highest solubility (2.75%), likely due to complete crystalline disruption and greater exposure of hydrophilic hydroxyl groups. Similar cold-water solubility (approximately 2%) has been reported for amorphous potato starches, with solubility increasing to ∼8% after incubation at 90 °C in previous studies (Kim et al., 2017). However, these values remain relatively low compared to those of typical cold water-soluble starches prepared by alkaline-alcohol treatment (Qian et al., 2019; Xu et al., 2025). At 60 °C, solubility increased across all samples, ranging from 1.80% to 3.94%. This enhancement likely reflects increased molecular mobility and loosening of hydrogen bonding networks, facilitating water penetration into the starch matrices. Notably, the increase was modest for MAGS-60 and MAGS-55, suggesting that most of the solubilization had already occurred at lower temperatures. These results align with previous findings (Liu et al., 2017; Singh & Singh, 2003), which demonstrated that the disruption of crystallinity and double helices enhanced hydrogen bonding between starch and water molecules.

The swelling power also exhibited a similar temperature-dependent trend. At 30 °C, NPS and MAGS-70 showed minimal swelling (2.70–2.80 g/g), while MAGS-60 and MAGS-55 showed significantly higher values (7.23 and 9.14 g/g, respectively), reflecting enhanced water uptake due to structural loosening. At 60 °C, all samples showed further increases, with swelling power reaching 13.70 g/g in MAGS-55. Interestingly, MAGS-60 displayed slightly higher swelling than MAGS-55, possibly due to excessive granule breakdown in the latter, which may have weakened its ability to retain water and form a cohesive matrix. In a comparative study by Majzoobi et al. (2015), pregelatinized potato starch showed a cold swelling capacity of 9.98 g/g at neutral pH, while granular cold-water-soluble starch exhibited 13.52 g/g, supporting our findings. This indicates that the extent of granule integrity plays a critical role in determining water retention capacity.

Although fully amorphous MAGS-55 and MAGS-60 exhibited enhanced cold-water solubility and swelling behavior compared with native starch, their hydration capacities remained lower than those of traditional cold-water-swelling starches. This difference may be attributed to the absence of alkaline treatment, which typically disrupts hydrogen bonding and promotes molecular dissociation, thereby increasing water accessibility and rapid swelling (Kaveh et al., 2020; Kim et al., 2017). Therefore, the functional performance of MAGS should be interpreted as moderate hydration enhancement rather than complete cold-water swelling.

### 3.7 Rheological properties

As shown in Fig. 3C and Table 4, the shear stress-shear rate behavior of all samples was well described by the power law model (τ = K·γⁿ), with a high coefficient of determination (R^2^ > 0.99). Interestingly, while the consistency coefficient (K) generally decreased with increasing amorphization up to MAGS-60, a sharp increase was observed for MAGS-55 (16.25 Pa·s). This contrast is particularly noteworthy because both MAGS-60 and MAGS-55 were fully amorphous based on DSC and birefringence analyses. SEM images indicate that MAGS-60 retained partially discrete granule-like frameworks despite internal amorphization, whereas MAGS-55 exhibited complete collapse into a continuous matrix. In MAGS-60, residual particle boundaries likely allow localized deformation and particle–particle slippage under shear, resulting in lower flow resistance. In contrast, the structural collapse in MAGS-55 may promotes the formation of a more continuous polymer-rich network in which starch chains are more extensively exposed and entangled. This reorganization may increases intermolecular interactions and may restrict molecular mobility under shear, thereby contributing to the sharp increase in K and the transition toward near-Newtonian flow. The flow behavior index (n) increased from 0.34 ± 0.02 in NPS to 0.95 ± 0.05 in MAGS-55, indicating a progressive reduction in shear-thinning behavior with increasing structural disruption. Conversely, the lower n values observed in NPS and MAGS-70 suggest stronger pseudo-plasticity may associated with retained granular or semi-crystalline domains. Similar trends in AGS systems have been reported by Fang et al. (2021), supporting the role of granule integrity and structural organization in determining rheological performance. Collectively, these results suggest that rheological behavior is governed not only by crystallinity loss but also by differences in granular topology and network continuity.

**Table 4:** Rheological parameters (power-law model) and digestion kinetics of MAGS prepared with different ethanol concentrations.

	Rheological properties			Digestion kinetics		
	K (mPa·s)	n	R^2^	C_∞_	*k* (×10^−2^)	R^2^
NPS	14.38 ± 0.60^ab^	0.34 ± 0.02^d^	0.99	19.76 ± 1.27^d^	1.47 ± 0.20^a^	0.94
MAGS-70	14.25 ± 0.39^ab^	0.38 ± 0.04^d^	0.99	43.94 ± 1.44^c^	1.51 ± 0.11^a^	0.98
MAGS-65	10.25 ± 0.99^cd^	0.52 ± 0.04^c^	0.99	64.75 ± 2.21^b^	1.53 ± 0.12^a^	0.98
MAGS-63	11.14 ± 0.82^bc^	0.62 ± 0.03^b^	0.99	72.05 ± 2.97^a^	1.66 ± 0.16^a^	0.97
MAGS-60	7.45 ± 0.28^d^	0.91 ± 0.02^a^	0.99	75.37 ± 3.14^a^	1.69 ± 0.17^a^	0.97
MAGS-55	15.58 ± 1.95a	0.95 ± 0.05a	0.99	75.55 ± 2.91a	1.70 ± 0.16a	0.97

*Values are expressed as mean ± standard deviation.

*Different letters within the same column indicate significant difference (*p* < 0.05).

The apparent viscosity at a given shear rate progressively increased with the degree of starch amorphization (Fig. 3D). Among all samples, MAGS-55 exhibited the highest viscosity across the entire shear rate range, reaching approximately 30 mPa·s at 1 s^−1^ and gradually declining to about 10 mPa·s at 100 s^−1^. This indicates a highly swollen and disordered starch structure that facilitates greater molecular entanglement and water binding. In contrast, NPS and MAGS-70 showed the lowest viscosity with relatively flat shear rate–viscosity curves, indicating limited swelling due to more crystalline structures. Banerjee and Kumar (2024) similarly proposed that heat treatment induces molecular rearrangements that increase shear resistance and viscosity due to enhanced molecular entanglement. The intermediate MAGS samples (MAGS-65, MAGS-63, and MAGS-60) displayed moderate viscosity levels, consistent with gradual increases in gelatinization and partial disruption of crystallinity. For instance, MAGS-60, which exhibited extensive but incomplete amorphization, showed a viscosity of ∼10 mPa·s at low shear rates, suggesting a balance between water absorption and structural resistance. These results suggest that microwave-ethanol treatment effectively modulates the rheological properties of starch by adjusting its granule structure and crystallinity, which in turn influences its viscosity, flow resistance, and processability. This behavior may be beneficial for systems requiring shear-responsive textures, such as sauces and pastes.

### 3.8 *In-vitro* digestibility

**Fig. 4:**
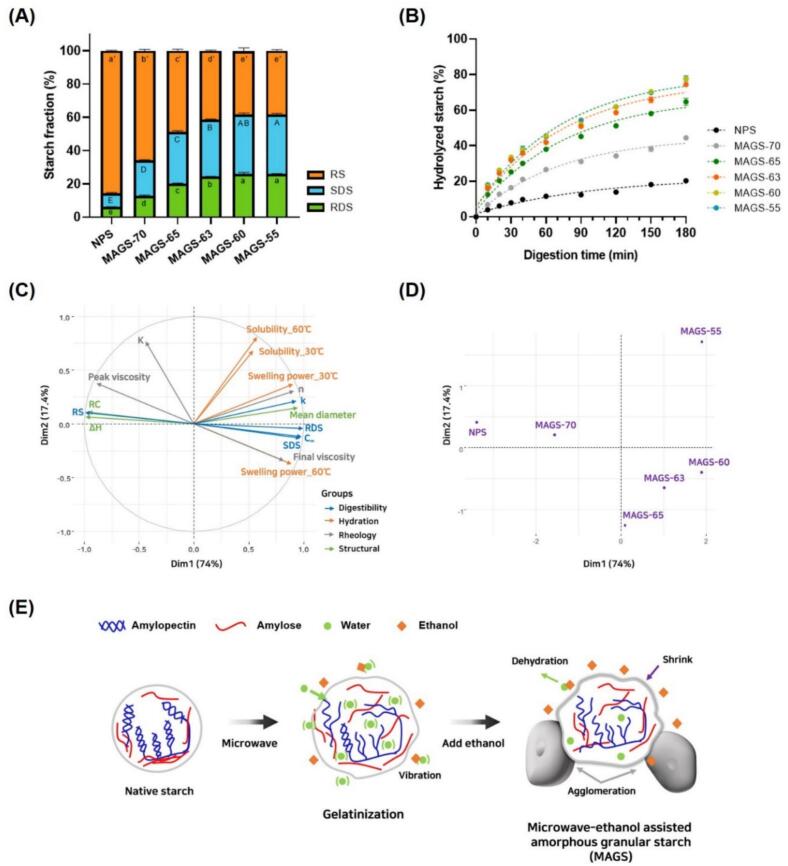
*In-vitro* digestibility (A), starch hydrolysis profiles (B), MFA variable map (C) and individual map (D), and proposed structural transformation mechanism (E) of MAGS.

To evaluate the intrinsic structural accessibility of native and microwave–ethanol–treated starches, the digestion assay was conducted without prior thermal gelatinization. Thermal pre-treatment would otherwise induce complete gelatinization and diminish structural distinctions introduced by the modification process. The digestibility characteristics of starch are crucial indicators of its nutritional and functional properties, particularly the RS content and enzymatic accessibility. In this study, MAGS exhibited significantly altered digestibility profiles compared with those of NPS. Native starch with its intact granular structure is predominantly classified as type RS1 due to its high RS content and low enzymatic accessibility. As shown in Fig. 4A, NPS had the highest RS content (85.67%) but the lowest RDS fraction (6.12%), indicating limited enzymatic accessibility. That is consistent with previous reports indicating RS values of 70–90% in NPS (Lee, Lee, & Lee, 2018). In contrast, MAGS samples exhibited markedly increased RDS and SDS fractions, accompanied by a substantial reduction in RS content. For example, MAGS-65, which had a gelatinization degree of 66.75% (based on DSC results), showed more than a 3-fold increase in RDS and SDS content, while the RS content decreased by approximately 63%. Notably, MAGS-63, which retained trace crystallinity, presented RDS of 24.44%, SDS of 58.59%, and only RS of 16.96%. Furthermore, MAGS-60 and MAGS-55, which lost all crystalline structures but exhibited differences in granule integrity showed no significant differences in starch fraction composition. Both samples displayed SDS contents exceeding 35%, while the RS content decreased to 38%. The increased SDS fraction can be attributed to the combined effect of partial amorphization and retained granule cohesion. Although crystalline lamellae were largely disrupted, the preservation of external granule structure can limit immediate enzyme diffusion, resulting in gradual hydrolysis rather than rapid conversion to RDS. Starch digestion is governed by multiple processes, among which enzyme diffusion into the granule is a key factor controlling the rate of hydrolysis (Wang, Luo, Zhang, He & Wang, 2014). This structural configuration is consistent with the elevated SDS content observed in MAGS samples. This observation aligns with previous studies reporting that starch systems with a balance between amorphous and ordered regions exhibit increased SDS content, as such intermediate structures provide partial enzymatic accessibility while maintaining structural resistance (Wang et al., 2025).

Application of the first-order kinetic model (Fig. 4B and Table 4) revealed that the equilibrium hydrolysis extent (C∞) increased from 19.76% (NPS) to 75.55% (MAGS-55), while the rate constant (k) rose from 1.47 × 10^−2^ to 1.70 × 10^−2^ min^−1^. This increase in C∞ corresponded well with the higher RDS and SDS fractions observed in MAGS samples, indicating that structural amorphization enhanced the overall extent of enzymatic hydrolysis, whereas variations in k were relatively minor. These findings confirm that microwave–ethanol treatment markedly enhances enzymatic hydrolysis through increased structural accessibility, consistent with earlier reports on AGS (Fang et al., 2021; Xu et al., 2025). Mechanistically, increased enzymatic accessibility can be associated with the amorphization and partial stabilization induced by the microwave–ethanol system. Rapid volumetric heating under dielectric polarization likely disrupts internal crystalline lamellae and short-range double helices, increasing enzyme-accessible regions within the granule (Oyeyinka et al., 2021). At the same time, the ethanol-rich phase limits excessive swelling and preserves partial granule morphology, enabling controlled amorphization without full gelatinization. This combination results in granules that are internally disordered yet externally cohesive, facilitating gradual enzyme penetration and sustained hydrolysis.

### 3.9 Multiple factor analysis

Multiple factor analysis (MFA) was employed to integrate structural, thermal, hydration, rheological, and digestibility parameters and to visualize their interrelationships. Dim1 explained 74% of the total variance and was primarily associated with the structural transition from semi-crystalline native starch to amorphous granular starch (Fig. 4C). Hydration-related variables, including solubility and swelling power at both temperatures, showed strong positive loadings along Dim1, indicating a close association between structural disorder and enhanced water interaction. Digestibility parameters (RDS, SDS, C∞, and k) were also closely aligned along the positive direction of Dim1, indicating that the reduction in crystalline order is closely associated with increased enzymatic susceptibility and a shift toward more digestible starch fractions. In contrast, relative crystallinity (RC) and gelatinization enthalpy (ΔH) were negatively correlated with Dim1, reflecting their association with preserved structural order.

The individual MFA (Fig. 4D) further illustrated the graded structural transformation across samples. NPS was positioned on the negative side of Dim1, consistent with its high crystallinity, limited swelling, and low digestibility. Samples treated with lower ethanol concentrations (MAGS-60 and MAGS-55) were positioned at the far positive end, reflecting pronounced amorphization, enhanced hydration properties, and markedly increased enzymatic hydrolysis. Intermediate samples (MAGS-70, MAGS-65, and MAGS-63) were distributed progressively along Dim1, indicating incremental modification of granule structure and functionality as a function of ethanol concentration. Collectively, these results confirm that microwave–ethanol treatment induces multiscale alterations in starch structure, hydration behavior, and digestibility.

### 3.10 Mechanism of starch modification by microwave-ethanol treatment

Fig. 4E presents a proposed mechanism describing the structural transformation of starch granules during microwave–ethanol treatment. Unlike conventional conductive heating, microwave irradiation enables volumetric dielectric heating through interactions between electromagnetic fields and polar molecules, primarily bound water. This process results in rapid localized energy deposition within the granule matrix, which may contribute to disruption of crystalline lamellae (Oyeyinka et al., 2021; Zhong et al., 2019). Native potato starch exhibits a semi-crystalline architecture composed of alternating crystalline and amorphous lamellae. Upon microwave–ethanol exposure, limited water availability in the ethanol-rich medium moderates excessive swelling while allowing internal structural disorder to develop. The absorbed microwave energy may weaken intra- and intermolecular hydrogen bonding within crystalline regions, promoting amorphization while partially preserving external granule morphology (Wei et al., 2025). This behavior differs from conventional alkaline-based amorphization, which typically involves high NaOH concentrations and prolonged treatment times and often leads to extensive molecular dissociation and granule erosion. In contrast, the present microwave–ethanol system reduced relative crystallinity from 21.81% to 3.20% within 1 min without chemical reagents. Under selected ethanol conditions (60–63%), substantial amorphization was achieved while retaining granular morphology, highlighting both structural and processing advantages over alkaline systems.

## 4 Conclusion

In this study, microwave–ethanol treatment provided a rapid and controllable method to modify the hierarchical structure of starch granules while preserving their overall morphology. The incorporation of ethanol effectively limited excessive gelatinization, enabling control over the extent of amorphization and structural disruption. Treatment with 60% ethanol promoted internal disordering of crystalline lamellae without compromising granule integrity, whereas lower ethanol concentrations resulted in complete collapse and gel-like structures. Multiscale analysis confirmed that microwave–ethanol-treated starches exhibited coordinated changes in crystalline organization, thermal behavior, rheological response, hydration properties, and enzymatic digestibility. The disappearance of typical pasting profiles and the shift toward near-Newtonian flow reflected substantial alterations in molecular interactions and hydration dynamics. Furthermore, the increase in rapidly and slowly digestible starch fractions, together with accelerated hydrolysis kinetics, demonstrates that the digestibility profile of starch can be modulated through solvent-assisted microwave treatment. Overall, ethanol concentration during microwave exposure serves as a key processing parameter governing the balance between granule integrity and internal amorphization. This solvent-assisted, time-efficient, and alkaline-free approach provides a practical route for producing amorphous granular starches with controlled structural and functional characteristics. Compared with conventional alkaline-based systems that require strong reagents and extended treatment times, this approach enables rapid structural modification under alkaline-free conditions while better preserving granular morphology.

## CRediT authorship contribution statement

**Seon-Min Oh:** Writing – original draft, Methodology, Conceptualization. **Seung-Hye Woo:** Formal analysis. **Min Kyung Park:** Validation, Formal analysis. **Joon-Young Jun:** Validation, Methodology. **Yun-Sang Choi:** Writing – review & editing, Supervision.

## Declaration of competing interest

The authors declare that they have no known competing financial interests or personal relationships that could have appeared to influence the work reported in this paper.

## Data Availability

Data will be made available on request.
